# Effect of interventions to reduce malaria incidence among military personnel on active duty: study protocol for a cluster randomised controlled trial of the impact of etofenprox-treated uniforms, permethrin-treated uniforms and DEET insect repellent

**DOI:** 10.1186/s13063-021-05801-9

**Published:** 2021-11-21

**Authors:** Daniel Msellemu, Amanda Ross, Lucky Temu, Irene Moshi, Lorenz Hofer, Charles Mwanziva, Yadon M. Kohi, Sarah J. Moore

**Affiliations:** 1 Environmental Health and Ecological Sciences Department, Ifakara Health Institute, Ifakara, Tanzania; 2 Swiss Tropical and Public Health Institute, Socinstrasse 57, 4051 Basel, Switzerland; 3 University of Basel, Petersplatz 1, 4003 Basel, Switzerland; 4 HJF Medical Research International (HJFMRI), Walter Reed Program, Tanzania, P.O. Box 13303, Dar es Salaam, Tanzania; 5 Tanzania People’s Defence Force, Magore Upanga, P.O. Box 9203, Dar es Salaam, Tanzania

**Keywords:** Malaria, Insecticide-treated clothing, Military uniforms, Etofenprox, Permethrin, Cluster randomised trial, CRT, DEET, Repellent

## Abstract

**Background:**

While there is strong evidence that bite protection methods such as permethrin-treated clothing and topical insect repellents are protective against insect bites, there are few studies assessing the impact on malaria infection. This study will estimate the protective efficacy of treated uniforms and DEET insect repellent on the incidence of malaria infection among military personnel in an operational setting. Permethrin-treated uniforms used with DEET lotion will be compared to etofenprox-treated uniforms with DEET lotion. The effect of DEET lotion will be estimated by comparing permethrin-treated uniforms with DEET or placebo lotion.

**Method:**

A cluster randomised double-blind placebo-controlled trial is planned to evaluate the effectiveness of the interventions on preventing malaria infections in soldiers on active duty at Mgambo National Service Camp in Tanga, Tanzania. The arms are (1) permethrin-treated uniform with 30% DEET liposome formula; (2) permethrin-treated uniform with placebo lotion; (3) candidate insect repellent system, i.e. etofenprox-treated uniform with 30% DEET liposome formula; and (4) placebo, i.e. untreated uniforms with placebo lotion. The primary outcome is the incidence of *Plasmodium falciparum* malaria infection detected by polymerase chain reaction (PCR) by active case detection using surveys every 2 weeks for 12 months. Rapid diagnostic tests will be used for the diagnosis of participants with symptoms.

The unit of randomisation will be combania: companies formed by recruits aged 18 to 25 years; combania do activities together and sleep in the same dormitory. Unequal randomisation will be used to optimise statistical power for the primary comparison between permethrin-treated uniforms with DEET and etofenprox-treated uniforms with DEET.

**Discussion:**

This trial will provide the estimate of the effects of permethrin with DEET compared to those of the new fabric treatment etofenprox with DEET and any additional effect of using DEET. The results will inform strategies to protect military personnel and civilians who have more outdoor or occupational malaria exposure than the general public.

**Trial registration:**

ClinicalTrials.govNCT02938975.

**Supplementary Information:**

The online version contains supplementary material available at 10.1186/s13063-021-05801-9.

## Administrative information

Note: the numbers in curly brackets in this protocol refer to SPIRIT checklist item numbers. The order of the items has been modified to group similar items (see http://www.equator-network.org/reporting-guidelines/spirit-2727-statement-defining-standard-protocol-items-for-clinical-trials/).

**Table:** 

Title (1)	Effect of Interventions To Reduce Malaria Incidence Among Military Personnel On Active Duty: study protocol for a cluster randomised controlled trial of the impact of etofenprox-treated uniforms, permethrin-treated uniforms and DEET insect repellent
Trial registration {2a and 2b}.	**Trial identifier:** Registration number NCT02938975 **Registry name:** ClinicalTrials.gov
Protocol version {3}	Issue Date: 30 Sept. 2020Version 06
Funding {4}	The trial is funded by the U.S. Armed Forces Pest Management Board (AFPMB). The Tanzania People’s Defence Force (TPDF) will provide the trial facilities, and the Ifakara Health Institute Quality Assurance (QA) Unit will provide trial oversight.
Author details {5a}	Daniel Msellemu*^1, 2, 3^, Amanda Ross ^2, 3^, Lucky Temu^5^, Irene Moshi^1^, Lorenz Hofer^2,3^, Charles Mwanziva^4^, Yadon M. Kohi^4^, Sarah J. Moore^1, 2, 3^ **Affiliations:** ^1^ Environmental Health and Ecological Sciences Department, Ifakara Health Institute, Tanzania ^2^ Swiss Tropical and Public Health Institute, Socinstrasse 57, 4051 Basel, Switzerland ^3^ University of Basel, Petersplatz 1, 4003 Basel, Switzerland ^4^ Tanzania People’s Defence Force, Magore Upanga, P.O. Box 9203, Dar es Salaam, Tanzania ^5^ HJF Medical Research International (HJFMRI), Walter Reed Program, Tanzania, P.O. Box 13303, Dar es Salaam, Tanzania
Name and contact information for the trial sponsor {5b}	Ifakara Health Institute **Plot 463, Kiko Avenue, Mikocheni B,** **P.O. Box 78373, Dar es Salaam,** **United Republic of Tanzania**
Role of sponsor {5c}	Overall program management, data management, statistical analyses and reporting. Data safety is overseen by an independent Institutional Review Board. Quality assurance is overseen by an independent Quality Assurance Unit.

## Introduction

### Background and rationale {6a}

People who live or temporarily stay away from permanent housing can be exposed to malaria vectors outdoors [1]. These people include military personnel on active duty [2, 3], residents and workers in the forests of South America [4] and Southeast Asia [5], migrants [6, 7] and displaced populations [8]. In addition to long-lasting insecticide-treated nets (LLINs) and indoor residual spraying (IRS), measures used to tackle vector-borne disease in these special populations include chemoprophylaxis, mass drug administration (MDA) [9] and mosquito bite prevention [10].

Means of bite prevention include repellents, which are applied to the skin (topical repellents) or to an area through volatilisation (spatial repellents) [11] and insecticide-treated clothing [12]. The most common active ingredient (AI) used to treat clothes, including uniforms, is permethrin [12]. Recently, etofenprox has become available. Etofenprox has a better human safety profile and is wash resistant up to 70 washes [13], providing prolonged protection. Permethrin and etofenprox function by irritating mosquitoes and other arthropods so that they do not bite through clothing [14]. DEET (*N*,*N*-diethyl-meta-toluamide) interferes with insect host location [15] and is the most effective available broad-spectrum topical insect repellent [16]. It has an excellent safety profile and has been in use for over 60 years [15]. It is applied directly to uncovered areas of the skin.

While there is very strong evidence that repellents and treated clothing are effective in reducing bites from biting arthropods [17], a recent systematic review found no evidence that topical repellents prevent malaria, insufficient evidence that spatial repellents prevent malaria and limited evidence that permethrin-treated clothing prevents malaria [18]. Two randomised control trials (RCTs) were included in the review; the evidence from them was deemed to be of low certainty due to the size and conduct of the studies. These studies were conducted in specific populations in Colombia (military personnel) and Pakistan (Afghan refugees). Insecticide-treated clothing may have a protective effect against clinical malaria caused by *Plasmodium falciparum* (risk ratio 0.49; *95% CI* (0.29–0.83)) and *P. vivax* (risk ratio 0.64; *95% C*I (0.40–1.01)) [18]. Therefore, World Health Organization (WHO) guidelines do not recommend insecticide-treated clothing for day-to-day use by the general population, although it may be beneficial as an intervention to provide personal protection against malaria in specific population groups [19].

In the case of military uniforms, the surface area of skin protected by DEET is small compared to that protected by clothing, limited to the hands, neck and face, and it is likely that the additional DEET provides only minimal additional protection. Furthermore, daily compliance with repellents as required for optimal disease prevention is rarely met, even for short periods of time [20]. It is therefore useful to estimate the protection provided by DEET in addition to treated uniforms to evaluate its utility and cost-effectiveness.

This study will evaluate the effectiveness on the incidence of malaria infection detected by polymerase chain reaction (PCR) of permethrin-treated and etofenprox-treated uniforms alone and in combination with DEET. We will also evaluate adherence to the interventions.

### Objectives {7}

#### Primary objective

To estimate the effect of etofenprox- compared to permethrin-treated uniforms by comparing etofenprox-treated uniforms (ETUs) plus DEET lotion compared to permethrin-treated uniforms (PTUs) plus DEET lotion on the incidence of *Plasmodium falciparum* malaria confirmed by polymerase chain reaction (PCR)

#### Secondary objectives

1. To estimate the protective efficacy of DEET lotion by comparing PTUs with DEET lotion to PTUs with placebo lotion on the incidence of PCR-detected *Plasmodium falciparum* malaria infection
2. To estimate the protective efficacy of PTUs plus placebo lotion compared to untreated uniforms and placebo lotion on the incidence of PCR-detected *Plasmodium falciparum* malaria
3. To estimate the protective efficacy of ETUs plus DEET lotion compared to untreated uniforms and placebo lotion on the incidence of PCR-detected *Plasmodium falciparum* malaria infection, i.e. the protection of etofenprox-treated clothing with DEET
4. To estimate the compliance of personnel with the system of tropical environment protection

### Trial design {8}

#### Study design

The trial is a four-arm cluster randomised double-blind placebo-controlled study. The unit of randomisation is the company, or *combania.* A combania is a military subsection of recruits who carry out all activities together and sleep in the same dormitory.

## Methods: participants, interventions and outcomes

### Study setting {9}

The trial will take place at Mgambo National Service Camp in Kiswahili Jeshi la Kujenga Taifa (JKT) military camp in Tanga region. Tanga is on the east coast of Tanzania, 250 km north of the commercial capital of Tanzania, Dar es Salaam. The climate is tropical, with temperatures of 21 to 33 °C during the hottest period (November to February) and 20 to 30 °C in the cooler period (May to August). The rainfall seasons are bimodal, with shorter rains from October to December and longer rains from March to May. The prevalence of malaria infection among recruits determined by a rapid diagnostic test (RDT) is about 30% [21].

### Eligibility criteria {10}

#### Study participants

Healthy recruits of the Tanzanian National Service Program JKT aged 18 to 25 who meet the trial inclusion criteria (Table 1) and who are enrolled into the trial upon written informed consent. Any participants who do not attend the screening for more than 2 months will be assumed to have dropped out. This may be due to deployment to another area or expulsion for misconduct.

**Table 1:** Inclusion, exclusion and withdrawal of trial participants

Inclusion criteria	Exclusion criteria	Withdrawal criteria
New recruits to national service JKT at Mgambo and do not use any malaria prophylaxis	**Already employed military personnel	Participants who wish to use malaria prophylaxis in parallel with intervention provided
Member of a *combania	Not assigned to a combania	Participants who do not reside in combania dormitories
Passes physical examination—no underlying health risks	Underlying health risks—does not pass the physical examination (No recruits to JKT are accepted if they have chronic health problems.)	Acquired illness or physical condition that makes a participant permanently not involved in military instructions
Recruits who are not pregnant	(Pregnant people are not recruited to JKT.)	Participant known to have become pregnant
Consents to participate	Does not consent to participate	Withdraws consent to participate

*Recruits are grouped into companies, or *combania*, which will be the level of randomisation

**Employed military personnel are combat ready and can be deployed at any time to any mission. National service members are available for at least a year and are not involved in combat missions

### Who will take informed consent? {26a}

Before enrolment, the aims of the study and study procedures will be explained to the participants, along with their right to withdraw from the study at any time. People who agree to enrol in the study will sign a consent form. Interviewers will be trained on the elements of informed consent and on the interviewing technique to respect the dignity and privacy of participants. Non-military staff will conduct all data collection to prevent potential coercion by military staff. Any person not willing to participate will be provided with standard untreated uniforms and will not be followed up.

### Additional consent provisions for collection and use of participant data and biological specimens {26b}

After PCR testing for *Plasmodium falciparum*, blood spots will be destroyed by incineration.

### Interventions

#### Explanation for the choice of comparators {6b}

The trial will use PTUs in combination with DEET as an active comparator to ETUs with DEET because PTU plus DEET is the existing personal protection method most widely used by the military globally. Untreated uniforms with placebo DEET will be a placebo comparator group to examine the absolute protective efficacy in the PTU-with-DEET and ETU-with-DEET study arms. This provides an opportunity to examine these two intervention systems simultaneously in a randomised control trial for the first time. The use of PTU without DEET will allow the evaluation of any additional protective benefit of using DEET.

#### Intervention description {11a}

Each participant will receive two uniforms, both treated according to the treatment arm. The uniforms will be impregnated with insecticide at Warmkraft–Pine Belt Processing Inc. Participants will wear one of the uniforms all day every day. The second uniform will be worn when the first is being washed. The insecticide in the uniforms will still be viable even after 50 washes [22–24].

##### PTU

No adverse health risks are known to be associated with wearing PTUs. Safety testing of permethrin has been ongoing since the 1970s, following its US Environmental Protection Agency (EPA) registration for use in a variety of applications, including food crops, animal feed crops, livestock, public health mosquito abatement programmes, pets and clothing [25]. Permethrin was first registered for use as a repellent on military clothing in 1990 and is the only insect repellent currently used for factory treatment of clothing. It is a broad-spectrum, non-systemic, synthetic pyrethroid insecticide that targets adults and larvae of many species of biting insects. The EPA has determined that wearing or coming in contact with PTUs for 250 days a year is unlikely to cause adverse health effects [12]. The objective is to provide 90% bite protection for at least 50 launderings, an objective easily met through factory treatment of the army combat uniform (ACU) with permethrin, which demonstrates 99 to 100% bite protection for up to 50 launderings [26].

##### ETU

No adverse health risks are known to be associated with wearing etofenprox-treated clothing. Etofenprox has now received EPA registration for use on military uniforms. The WHO dermal risk assessment for etofenprox is based on a no-observed-adverse-effect level (NOAEL) of 2100 mg/kg/day, which is four times greater than that of permethrin. Uniforms will be factory treated with 0.9% etofenprox. When compared to untreated control fabric, etofenprox-treated fabric demonstrated greater than 90% bite protection after 3 washes [27] and has already been approved for use by US Army personnel.

##### DEET

Each participant from a combania that receives DEET will be given a 120-mL container of 30% DEET repellent (Lipo DEET) for topical application, and the container will be replaced every 2 weeks. Combania in placebo arms will be given 120 mL of placebo lotion. The DEET lotion and its placebo will be prepared by Sawyer Ultra 30. Participants will be instructed to apply the repellent topically twice daily: once in the morning and once in the evening. One application of Lipo DEET protects for up to 12 h and has a pleasant odour and non-greasy feel on the skin. This liposome-based repellent is the newest advance in insect repellent technology. Like the earlier generation of polymer-based controlled release systems, the liposome envelops the active ingredient, DEET, and slowly releases it as needed, thereby extending the repellent’s effectiveness. DEET was selected for this study because it has been extensively tested for safety and toxicity for human use [28–30] and for its efficacy against a broad variety of arthropod vectors [31–33]. DEET was first registered in 1957 and has an excellent safety profile [34, 35].

### Criteria for discontinuing or modifying allocated interventions {11b}

There are no plans to discontinue or modify the interventions. Follow-up is planned for 12 months to include the bimodal rainfall seasons, which affect mosquito abundance and malaria transmission. It is likely to be difficult to demonstrate futility without the full data set due to year-to-year variation in the seasonal pattern. Issues of safety are not expected because the products are already approved. In the case of severe adverse reactions, individual participants will be withdrawn from the trial.

### Strategies to improve adherence to interventions {11c}

During the consent procedure and the distribution of uniforms and DEET lotion, participants will be introduced to the importance of adherence. Containers of DEET lotion will be replaced every 2 weeks when the participants are tested for active malaria infection. Used containers will be weighed to calculate the average daily application. Since uniforms are worn every day at the camp and recruits are not allowed to wear any other outfit, adherence to uniform wearing is expected to be extremely high. Every 2 weeks, when participants are tested for active malaria infection, they will complete a questionnaire about activities that may affect malaria exposure, such as emergency home leave, and about adherence to uniforms and lotions. Participants who have left the camp during the 2 weeks before testing will be removed from follow-up for those 2 weeks.

### Relevant concomitant care permitted or prohibited during the trial {11d}

Participants are advised to refrain from self-prescription of any medication. All antimalarials will be dispensed to only participants with positive RDT.

### Provisions for post-trial care {30}

**Fig. 1:**
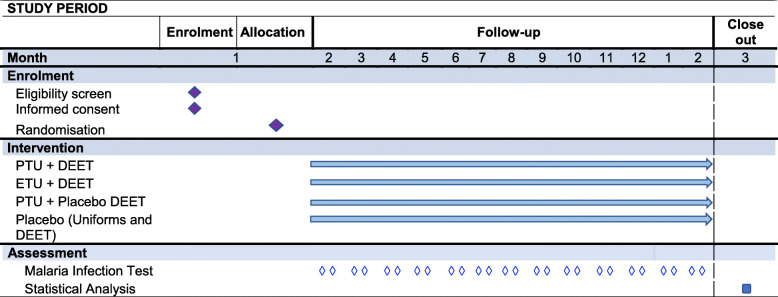
Schedule of events for the trial

All recruits who will give their consent to participate will be insured for a period of 14 months (Fig. 1). The insurance will cover the 12 months of trial follow-up and 2 months after the trial ends on health issues that arise as a direct consequence of trial participation. During the trial, any participant who experiences a serious adverse event related to the study intervention will be transported to Bombo Regional Hospital. Referral to the regional hospital is to provide ancillary needs that may otherwise arise during trial participation.

The insurance will pay for ancillary care. This trial is considered to be of a minimum risk to participants and the form of compensation to be provided is treatment of participants who are harmed from trial-related incidents.

### Outcomes {12}

#### Primary outcome

The primary outcome is the incidence of *Plasmodium falciparum* infection as determined by active case detection through quantitative polymerase chain reaction (qPCR) in blood samples taken every 2 weeks and by passive case detection at the camp clinic in the case of illness. Personal travel by participants will be assessed every 2 weeks with brief questionnaires to estimate time away from the camp. Time at risk will exclude a 14-day period post-travel and a 14-day prophylactic period following any malaria treatment. Malaria infections will be detected by qPCR; this method detects malaria infections below the limit of detection (LOD) of rapid diagnostic tests (RDTs), although RDT will be used at the point of care for any participants symptomatic for malaria. We will perform the PlasQ qPCR assay as described previously [36]. This triplex TaqMan chemistry-based qPCR assay detects the pan-*Plasmodium* 18S rRNA sequence (PSpp18S) [37, 38] and the *P. falciparum*–specific acidic terminal sequence of the var genes (PfvarATS) [39]. The human RNaseP (HsRNaseP) gene serves as an internal control to evaluate the quality of DNA extraction and qPCR amplification. After finger-pricking by a study nurse, a blood sample of 200 μL of whole blood is drawn into an EDTA-containing collection tube (Greiner, MiniCollect®); 180 μL of the whole blood will be mixed with an equal volume of DNA/RNA Shield™ (Zymo Research, Irvine, CA, USA) and incubated at room temperature for 30 min before storage at –20 °C. On a weekly basis, samples will be packed with liquid nitrogen in a thermo-col container and transported under cold chain to the qPCR facility. A temperature long will be used on the storage at the field facility, during transport and at the qPCR facility. Upon arrival, DNA will be extracted from the samples using Quick-DNA™ MiniPrep kits (Zymo Research, Irvine, CA, USA), following the manufacturer’s guidelines. Amplification and qPCR measurements will be performed using the Bio-Rad CFX96 Real-Time PCR System (Bio-Rad Laboratories, Hercules, CA, USA). All qPCR runs will be performed with a no-template control (NTC) and with *P. falciparum* NF54 DNA as a positive control.

### Participant timeline {13}

#### Sample size {14}

The sample size calculations are based on the comparison of the incidence of malaria infection between the trial arms. The primary comparison is between ETU + DEET and PTU + DEET. An expected total of 1500 recruits per year form combania with a mean of 40 recruits each. We expect roughly 37 combania available for randomisation. A combania is a military subsection of recruits who carry out all activities together and sleep in the same dormitory. The dormitories can accommodate 30 to 50 recruits. The trial is randomised by combania because of the risk of contamination between recruits in the same company if the trial were individually randomised and because it is expected that a whole company will use the same interventions in practice in a “real world” use scenario.

The outcome is the incidence of malaria infection determined by PCR every 2 weeks. As a baseline estimate (with neither treated uniform nor repellent), the most recent available incidence of malaria infection in Mgambo camp among recruits is 0.68 per person per year in 2015 [40].

The sample size was estimated using a simulation of 1000 trials using generalized linear mixed effects models written in R statistical software version to estimate the power and the precision of the estimated incidence rate ratio (IRR) (the precision is measured here by the expected width of the confidence interval). We assumed a Poisson distribution for the incidence of malaria infections among recruits and a lognormal distribution of the combania cluster effects.

An assumed efficacy of 50% for PTU + DEET with a compliance of 80% would lead to an incidence rate of 0.41 infections per person per year. An assumed efficacy of 70% for ETU + DEET and compliance of 80% would lead to an incidence rate of 0.27 and an IRR of 0.66. With 12 clusters each of 40 recruits and 12 months of follow-up, we would have 89% power to detect this difference as significant and be able to estimate the IRR with a confidence interval of 0.49 to 0.88. After exploring different scenarios for unequal cluster randomisation to maximise power, we will randomise 12 combania (about 480 participants) for each arm of the primary comparison (PTU + DEET vs. ETU + DEET) and 6 combania (about 240 participants) to each of PTU + placebo lotion and untreated uniform + placebo lotion.

### Recruitment {15}

The Mgambo JKT camp enrols 2000 recruits in every annual intake. We aim to recruit at least 1500 to the study. If new recruits join an existing combania in the trial, they will be approached for enrolment.

### Assignment of interventions: allocation

#### Sequence generation {16a}

Randomisation of the combania will be carried out by an independent statistician using a random number generator in R or Stata.

#### Concealment mechanism {16b}

Manufacturers of the uniforms and repellents will prepare active and placebo uniforms and repellent and placebo lotions in identical containers identifiable by a 6-digit code sewn into the uniform or applied on the lotion bottle. Each combania will be allocated an identification code. There will be one code per combania, to limit the effect of accidental unblinding, and a separate code for additional untreated uniforms and placebo DEET, in case of adverse events.

#### Implementation {16c}

The combania will be allocated to the intervention by an independent statistician, who will carry out the randomisation and retain the codes. This statistician will not be further involved in the trial. The code master list will be provided to a site manager by the statistician using email to ensure concealment. Uniforms and lotion will be assigned to combania by the study site manager following the code master list.

### Assignment of interventions: blinding

#### Who will be blinded {17a}

In this double-blinded placebo-controlled trial, the participants, investigators and study statistician will be blinded to the allocation of interventions by a combination of intervention codes and combania codes.

#### Procedure for unblinding if needed {17b}

In the advent of a severe adverse event thought to be connected to the intervention, the recruit will be allocated untreated uniforms. The time contributed to the study by a person who experienced an adverse event will be included in the intention-to-treat analysis. Unblinding of the participant will be carried out if necessary to allow the study doctor to provide appropriate care. Only the study doctor will be informed of the person’s intervention allocation; other participants and investigators will remain blinded. The IHI Institutional Review Board will be informed of the adverse event.

### Data collection and management

#### Plans for assessment and collection of outcomes {18a}

Data collection will be via questionnaires “Additional file 1” loaded onto tablet computers and uploaded every 2 weeks via mobile phone network to the IHI server, where the data will be checked and verified monthly by the study investigators.

Participants who are symptomatic (fever, defined as axillary temperature ≥ 38 °C; chills; headache; nausea and vomiting; muscle pain; fatigue) will be tested for *Plasmodium* infections using malaria rapid diagnostic tests (mRDTs) at point of care for clinical diagnosis. RDT data will also be uploaded in real time from tablet computers. During active case detection, every 2 weeks, a blood spot will be taken on Whatman filter papers [41] for analysis by real-time PCR [42] at Ifakara Health Institute. Each sample (RDT, filter paper and blood slides) will be labelled with participant identification number, combania and date. Treatment will be offered to all malaria-infected participants, and all treatment details will be filled in and added to the patient file.

Data from PCR analysis of blood spots taken to capture subpatent malaria cases will be entered into tablet computers at the laboratory for inclusion in the study data set. Data will be checked for quality before incorporation into the central database.

#### Plans to promote participant retention and complete follow-up {18b}

Participants in this trial are military recruits who are expected to stay in the camp for at least 2 years. The trial is designed to conclude before the end of voluntary participation in national service. However, new recruits joining an existing combania will be asked if they wish to enrol and recruits who leave the camp will not be followed up. The time when the recruits are on leave will not be included in the period of follow-up.

#### Data management {19}

Data will be collected using tablet computers and uploaded weekly to the IHI server. Access to the data will be password controlled. Data cleaning will be conducted weekly and will incorporate checking for outliers, unusual values, inconsistencies and missing entries. This will be followed by fixing structural errors, removing duplicates, correcting unusual values for which a true value can be located, and removing unusual values for which a true value cannot be located [43].

To minimise data entry errors, questionnaires and data sheets on tablet computers will be programmed to give warnings when values outside of the expected range are entered, when the type of value entered is incorrect (for example, a numeric value rather than text), when a mandatory field is omitted and when identical values are entered.

#### Confidentiality {27}

To maintain participants’ confidentiality, data will be pseudonymised before analysis. Participants will be identified not by name but by codes known only to the researchers. The trial will report aggregate findings at combania and study arm levels but not individual findings.

#### Plans for collection, laboratory evaluation and storage of biological specimens for genetic or molecular analysis in this trial/future use {33}

Not applicable; blood samples will be used only for malaria diagnosis.

### Statistical methods

#### Statistical methods for primary and secondary outcomes {20a}

The primary analysis will be an intention-to-treat analysis. A per-protocol analysis will also be performed where person-time at risk will be adjusted to exclude 2 weeks of follow-up of participants who (1) have not complied with the lotion, (2) have taken antimalarials or (3) have travelled outside the camp for personal reasons within the previous 2 weeks. Statistical analysis will be done in Stata (StataCorp, College Station, TX, USA) and R (The R Foundation for Statistical Computing, Vienna, Austria).

#### Defining compliance

Compliance with the intervention will be estimated as the proportion of 2-week time intervals where the participant was compliant and estimated by study arm and over time. The JKT recruits must wear uniforms and will be provided with two treated uniforms. They have no alternative clothing, leading to an expected 100% compliance; compliance with clothing will not be monitored. Participants’ repellent bottles will be weighed twice weekly to estimate application rates. If a bottle contains more than 50% of the required dose, the participant will be recorded as non-compliant.

#### Descriptive analysis

Baseline characteristics of participants will be summarised by the study arm using appropriate measures of centrality and variability such as proportions, the mean and standard deviation or median and 90% central range, depending on the distribution. Clustering by combania will be considered throughout.

#### Estimates of protective efficacy

The primary outcome is the incidence of new malaria infections identified by qPCR period per person-month at risk. The incidence rate ratio (IRR) to compare study arms will be estimated using a regression model with combania as a random effect to account for the clustering. Initially, we will adopt a Poisson or negative binomial model, depending on the data distribution. The effect of the intervention over time will be investigated using interaction terms.

An unadjusted estimate will be presented, as well as an estimate adjusted for factors that are identified a priori to be potential confounders (age, region of origin, body surface area, night-time activity, use of bed nets, frequency of uniform washing).

The results will be presented as incidence rate ratios with 95% confidence intervals and *p*-values. The protective efficacy of each intervention will also be estimated as (1 – IRR) × 100%.

#### Interim analyses {21b}

An interim analysis would be unlikely to stop the trial for futility or overwhelming intended benefit because the interventions are already approved products with tested mosquito bite prevention and because the number of malaria infections may increase in tandem with conditions for mosquitoes at irregular times in the year. However, if during the trial unusual weather affects malaria transmission intensity in the area resulting in decreased malaria transmission and if an incidence substantially of lower than 0.68 is observed in the control arm, then the study may be extended to capture another malaria transmission season. An interim analysis at 6 months will determine only the number of malaria infection incidents detected and preserve the blinding of the investigators and study staff.

#### Methods for additional analyses (e.g. subgroup analyses) {20b}

We do not plan any subgroup analysis.

#### Methods in analysis to handle protocol non-adherence and any statistical methods to handle missing data {20c}

We expect that there will be little missing data since there will be active follow-up during the trial. For all analyses, participants will be included for all time points where their data is not missing. If there is missing data, we will conduct sensitivity analyses to check that the calculations are not affected.

#### Plans to give access to the full protocol, participant-level data and statistical code {31c}

The project database containing entomological and epidemiological data will be de-identified based on local IRB requirements. The de-identified data and accompanying metadata in the Ifakara Health Institute data repository will be made available on reasonable request.

### Oversight and monitoring

#### Composition of the coordinating centre and trial steering committee {5d}

Not applicable. It is not a multicentre trial. Also, trials with minimal risks to participants do not necessarily require a data monitoring committee.

#### Composition of the data monitoring committee, its role and reporting structure {21a}

The trial is intended to examine interventions that reduce mosquito bites; permethrin, etofenprox and DEET are known already to have excellent human safety profiles [13, 15]. Trials with minimal risks to participants do not necessarily require a data monitoring committee (DMC) [44]. The trial does not examine prolonging of individual life or reduction of risk of a major adverse health outcome as do trials comparing mortality or morbidity of participants. Therefore, the trial will be minimal risk. However, the Ifakara Health Institute Institutional Review Board (IRB) will provide oversight through annual reports. The IRB comprises epidemiologists, statisticians, entomologists, social scientists and community leaders.

#### Adverse event reporting and harms {22}

The team physician will determine the relationship between any adverse event and the intervention. All adverse events will be treated as clinically indicated, and subsequent treatment will be recorded. If necessary, participants will be referred for special care at the local hospital. Study clinicians who determine that an AE/SAE (adverse event or severe adverse event) is intervention related and that stopping intervention is clinically indicated will stop the study intervention. All adverse events will be reported to the IRB. Adverse events will be recorded, and descriptive analysis of adverse events will be reported as part of the publication arising from the trial.

#### Frequency and plans for auditing trial conduct {23}

The trial will be overseen by the IHI Quality Assurance Unit, which will audit the trial protocol and all critical phases, i.e. enrolment, allocation of intervention, the study report and study archive.

#### Plans for communicating important protocol amendments to relevant parties (e.g. trial participants, ethical committees) {25}

The trial team is responsible for all amendments to the protocol. The fundamental amendments to be reported will include the change to the protocol that may modify study objectives, study procedures, inclusion criteria, sample sizes, trial outcome, analysis plan and blinding and other changes which may impact on the conduct of the study by either affecting the safety of trial participants or the scientific validity, scope or ethical thoroughness of the trial or bringing any potential benefit of the participants. Any of these amendments will be agreed upon by the study team and be approved by the IHI IRB and the National Institute of Medical Research (NIMR-Tanzania) and changes will be updated into the trial register. In addition, the IHI Quality Assurance Unit will be notified. When an old protocol is updated to a newer version, the new version of the protocol gets another number that identifies the protocol version and the date. This will help to track histories of amendments and marks the latest protocol version.

### Dissemination plans {31a}

Final trial results will be disseminated to stakeholders at the Tanzania People’s Defence Force (TPDF) and the scientific community via publication of study findings in open-access, peer-reviewed journals and international conference forums. In addition, yearly progress reports will be distributed to key stakeholders. All researchers and technical staff who contribute to the research will be authors on any publications arising from the research as per the Vancouver Protocol. The results of this trial are intended to be disseminated regardless of the magnitude or direction of effect.

## Discussion

Better ways to protect military personnel from malaria on active duty are required. Findings from the proposed trial will provide knowledge to improve malaria prevention among military personnel. We will provide information on which combination of interventions is most effective and may reduce unnecessary costs to institutions, such as the cost of topical repellents. Identifying factors for non-adherence may improve the design of future bite prevention interventions.

The trial will evaluate the impact of interventions in an active duty environment. The results may be relevant to other occupations with exposure to an increased risk of mosquito bites and vector-borne disease, including forest workers, guards and miners, loggers and rubber tappers [45]. If beneficial, other potential users could include visitors to endemic regions [46], relief workers [47] and refugees [48].

Insecticide-treated clothing may also protect wearers from day-biting mosquitoes, such as *Aedes aegypti*, which are currently increasing rapidly in Tanzania [49], as well as other arthropods that engage in nuisance biting. This trial will not test efficacy against dengue but may generate data needed for Tanzanian registration of insecticide-treated clothing that could have benefits for multiple vector-borne diseases. Etofenprox has not previously been put into a trial that estimates efficacy in combination with another repellent. Due to low toxicity and environmental safety [50], vendors may find a new niche in the community for impregnating clothes with the repellent.

The study involves a group of young people who, because they must wear their uniform as part of their job, will be more likely to comply effectively with the intervention than would the general population. While this is necessary to determine the true effect of insecticide-treated clothing to reduce disease under controlled conditions, the results may overestimate the effectiveness of the intervention for the general population. However, it is likely that compliance among military personnel will be similar to that among other users whose occupations require them to wear uniforms, such as guards, miners and nurses. This may influence generalizability of the findings, as the effectiveness of any personal protection method is dependent on compliance. Data generated from the comparison of the intention-to-treat and per-protocol analyses [51–53] will give useful information about compliance among the troops.

## Conclusion

Interventions that are protective when vectors are active and when humans are away from their houses need to be developed [54, 55]. This study will estimate the impact of insecticide-treated clothing and DEET lotion on reducing malaria infection transmission in military personnel.

## Trial status

Protocol version: BIT014_URCT_PR_v12

Protocol date 30/09/2020

Participant recruitment has not begun.

## Supplementary Information

**Additional file 1:.** Follow Up Questionnaire English

## Abbreviations

ACU: Army combat uniform
BIT: Bagamoyo Insecticide Testing
CO: Commanding officer
DEET: *N*,*N*-Diethyl-meta-toluamide
DMSNS: Director of Medical Services National Service
DNA: Deoxyribonucleic acid
DWFP: Deployed War-Fighter Protection
EDTA: Ethylenediaminetetraacetic acid
EPA: Environment Protection Agency (US)
ETU: Etofenprox-treated uniforms
GLMMs: Generalized linear mixed models
HQ: Headquarters
HsRNaseP: Human ribonuclease P
IHI: Ifakara Health Institute
IRB: Internal Review Board
IRR: Incidence rate ratio
JKT: Jeshi la Kujenga Taifa (National Service)
LOD: Limit of detection
MDA: Mass drug administration
NIMR: National Institute of Medical Research (Tanzania)
NOAEL: Non-observed adverse effect level
NTC: Non-template control
ORP HRPO: Office of Research Protections Human Research Protection Office
PCR: Polymerase chain reaction
PfvarATS: *Plasmodium falciparum* variant ATS
PR: Protocol
PTU: Permethrin-treated uniform
qPCR: Qualitative PCR
QC: Quality control
RNA: Ribonucleic acid
RDT: Rapid diagnostic test
RNaseP: Ribonuclease P
TPDF: Tanzania People’s Defence Forces
USAMRMC: United States Army Medical Research and Materiel Command
