# Supplementary material for: Effect of interventions to reduce malaria incidence among military personnel on active duty: study protocol for a cluster randomised controlled trial of the impact of etofenprox-treated uniforms, permethrin-treated uniforms and DEET insect repellent
Source: Trials. 2021 Nov 21;22:825. doi: 10.1186/s13063-021-05801-9 (PMC8607639; doi:10.1186/s13063-021-05801-9)
Supplement: Supplementary file 1 — Additional file 1:. Follow Up Questionnaire English [file 13063_2021_5801_MOESM1_ESM.doc]

# Appendix 2.

Follow Up Questionnaire English

Treated Military Uniform for Malaria Prevention

Introduction: Hello my name is “………………………………………………………….…..” I am from IHI

Do you agree to take part in the questionnaire? Yes - complete informed consent No – STOP

INFORMED CONSENT OBTAINED: [__] Yes [__] No – STOP

*To be filled in before the interview*

- 1. Interviewee unique ID |____|____|____|____|____|____|____|
  2. Interviewee force number |____|____|____|____|____|____|____|

0.3 Date of interview |____|____| / |____|____| / |____|____|____|____| (Day/Month/Year)

0.4 Name of the platoon |___________________________________________________________|

0.5 Platoon Identification number |____|____|____|____|____|____|

0.6 Name of the platoon commander |____________________________________________________|

| Section II: Malaria infection | | | |
| --- | --- | --- | --- |
| Qn # | *Questions and filters* | *Coding category* | *Answer (enter coding categories*) |
| 7 | Did you have malaria in the past two weeks  **If “NO” go to Qn 12** | 1…. Yes | |__|__| |
|  |  | 0…. No | \|__\|__\| |
|  |  |  |  |
| 8 | Did you test for malaria in the last two weeks RDT Results?  **If tested with RDT skip Qn 9** | 1…. Yes | |__|__| |
|  |  | 0…. No |  |
|  |  | Results (1) Positive  (0) Negative | \|___\| |
|  |  |  |  |
| 9 | Did you test for malaria in the last two weeks?  BS-MP Results | 1…. Yes | |__|__| |
|  |  | 0…. No |  |
|  |  | Results (1) Positive  (0) Negative | \|___\| |
|  |  |  |  |
| 10 | Did you take any antimalarial medication? | 1…. Yes | |____| |
|  |  | 0…. No | \|____\| |
|  |  |  |  |
| 11 | Name of the antimalarial you used  **Do not probe** | Name of the antimalarial | |________________| |
|  |  |  |  |
| 12 | Are you under any medication?  **If “YES” name the drug used in the last two weeks** | 1…. Yes  0…. NO | |____| |
|  |  |  | \|____\| |
|  |  |  | \|________________\| |
|  |  | | |
| Section II: Malaria Exposure at the camp | | | |
| Qn # | *Questions and filters* | *Coding category* | *Answer (enter coding categories*) |
|  |  |  |  |
| 13 | Did you sleep under the bed net last night? | 1… Yes | |___| |
|  |  | 0…. No | \|___\| |
|  |  |  |  |
| 14 | Do you use any mosquito control method like mosquito coil, sprays, topical repellant like DEET etc? | 1…. Yes | |___| |
|  |  | 0…. No |  |
|  |  |  |  |
| 15 | How many nights were you on guard post in the last two weeks?  **Write number of nights** | Total number of nights at guard post | |__|__| |
|  |  |  |  |
| 16 | Were you involved with other night duties in the past two weeks?  **If Yes: How many days** | 1… Yes | |___| |
|  |  | 0… No |  |
|  |  | How many days | \|__\|__\| |
|  |  |  |  |
| 17 | How many nights did you spend away from the camp on duty?  **Write number of nights** | Total number of nights away from camp.  **If not fill in |0|0|** | |__|__| |
|  |  |  |  |

| Section III: Activities that compromising adherence | | | |
| --- | --- | --- | --- |
| Qn # | *Questions and filters* | *Coding category* | *Answer (enter coding categories)* |
| 18 | Did you have any illness or injury in the past two weeks?  **If “No”: go to Qn 20** | 1. Yes | |__|__| |
|  |  | 0. NO |  |
|  |  |  |  |
| 19 | How many days were you on the sick bed in the last two weeks |  | |__|__| |
|  |  |  |  |
| 20 | How many nights did you spend away from the camp for personal reasons? |  | |__|__| |
|  |  | Number of nights |  |
|  |  |  |  |
| 21 | How many times did use your uniform in the last two weeks?  **If less that 7 nights ask; Why?** | Number of nights | |__|__|  |__________________| |
|  |  |  |  |
|  |  |  |  |
| Section IV: Activities that weakens repellant | | | |
| Qn # | *Questions and filters* | *Coding category* | *Answer (enter coding categories)* |
| 22 | What time do you wash? | Times in 12 hrs format | |__|__|: |__|__| |
|  |  |  |  |
| 23 | What time do you go to bed? | Times in 12 hrs format | |__|__|: |__|__| |
|  |  |  |  |
|  |  |  |  |
| 24 | What time do you apply the repellant? | Times in 12 hrs format | |__|__|: |__|__| |
|  |  |  |  |
|  |  |  |  |
| 25 | Did you wash your combat this week?  **If yes; How many times?** | 1. Yes | |___| |
|  |  | 0. No |  |
|  |  | Numbers of times washed | \|__\|__\| |
|  |  |  |  |
| 26 | Have you moved into a different platoon in the last two weeks | 1…. Yes | |___| |
|  |  | 2…. No |  |
|  | **If Yes; How many times?** | Write number of times | |__|__| |
|  |  |  |  |
| 27 | How many times did you not apply your repellant last week?  **If less than 14 times ask why?** | Number of times | |__|__| |
|  |  | **Tick that apply** |  |
|  |  | I forgot | \|___\| |
|  |  | I was away | |___| |
|  |  | I did not like it | |___| |
|  |  | It doesn’t work | \|___\| |
|  |  | I don’t need it | |___| |
|  |  | It was finished | |___| |
|  |  | Time was not enough | \|___\| |
|  |  | Other reasons (mention) | |___________________| |
|  |  |  |  |
| 28 | How many times did you not wear your uniform in the last week?  If less than 14 times ask why? | Number of times | |__|__| |
|  |  | **Tick that apply** |  |
|  |  | I do not want | |___| |
|  |  | It is tight | |___| |
|  |  | I did not like it | |___| |
|  |  | It doesn’t work | |___| |
|  |  | I gave it to some one | |___| |
|  |  | I get sick | |___| |
|  |  | It is too hot | \|___\| |
|  |  | Other reasons (mention) | |____________________| |
